# Engineered mesenchymal stem/stromal cells against cancer

**DOI:** 10.1038/s41419-025-07443-0

**Published:** 2025-02-19

**Authors:** Yuzhu Shi, Jia Zhang, Yanan Li, Chao Feng, Changshun Shao, Yufang Shi, Jiankai Fang

**Affiliations:** 1 The Third Affiliated Hospital of Soochow University, Institutes for Translational Medicine, State Key Laboratory of Radiation Medicine and Protection, Suzhou Medical College of Soochow University, Suzhou, Jiangsu 215123 China; 2 Department of Basic Medical Sciences, Changzhi Medical College, Changzhi, Shanxi 046000 China; 3 Department of Experimental Medicine and Biochemical Sciences, TOR, University of Rome “Tor Vergata”, Rome, 00133 Italy; 4 Shanghai Institute of Nutrition and Health, Chinese Academy of Sciences, Shanghai, 200025 China

**Keywords:** Mesenchymal stem cells, Targeted therapies

## Abstract

Mesenchymal stem/stromal cells (MSCs) have garnered attention for their potential in cancer therapy due to their ability to home to tumor sites. Engineered MSCs have been developed to deliver therapeutic proteins, microRNAs, prodrugs, chemotherapy drugs, and oncolytic viruses directly to the tumor microenvironment, with the goal of enhancing therapeutic efficacy while minimizing off-target effects. Despite promising results in preclinical studies and clinical trials, challenges such as variability in delivery efficiency and safety concerns persist. Ongoing research aims to optimize MSC-based cancer eradication and immunotherapy, enhancing their specificity and efficacy in cancer treatment. This review focuses on advancements in engineering MSCs for tumor-targeted therapy.

## Facts

- MSCs possess tumor-homing abilities and exhibit low immunogenicity.
- MSCs can be engineered to deliver therapeutic agents, such as proteins, microRNAs, and drugs, directly to the tumor microenvironment.
- Significant advancements have been made in engineering MSCs to enhance their efficacy in cancer treatment.

## Open Questions

- How can the targeting efficiency and therapeutic delivery of MSCs be further optimized?
- What are the long-term safety and efficacy profiles of engineered MSCs in clinical settings?
- How do MSC-based therapies compare with other emerging treatments, such as immune checkpoint inhibitors and CAR-T cell therapy, particularly regarding cost-effectiveness, patient outcomes, and long-term benefits?

## Introduction

Mesenchymal stem/stromal cells (MSCs) were first discovered in the bone marrow stroma in 1967 and identified as “colony-forming unit fibroblasts” [1]. Subsequently, it has been found that MSCs are distributed throughout nearly all types of tissue [2], including, but not limited to, the umbilical cord (UC) [3], adipose tissue (AD) [4, 5], and dental pulp (DP) [6]. As one of the most widespread cell types in the human body, MSCs exhibit an exceptional capacity to differentiate into multiple mesenchymal cell lineages, such as adipocytes, osteocytes, and chondrocytes [7, 8]. MSCs have elicited considerable interest due to their immune privilege, specifically low immunogenicity (absence of MHC-I expression and nearly negligible expression of MHC-II on the surface) [9].

Quality control standardization of MSC treatment may accelerate the advancement of MSC clinical applications. Since 2006, the International Society for Cell and Gene Therapy and the MSC Scientific Committee have promoted the standardization of MSC therapy in both preclinical researches and human translational studies [10–12]. The quality control explicitly defines the identification, purity, potency, proliferative capacity, genomic stability, and microbiological testing of MSCs. By the end of 2023, over 1000 MSC-based clinical trials were registered with *clinicaltrials.gov* to confirm the feasibility and effectiveness of MSC therapies for various diseases. MSCs have been successfully applied to treat inflammatory diseases and organ injuries, such as graft versus host disease and systemic lupus erythematosus [13, 14], due to their ability to migrate to sites of tissue injury and participate in repair [15]. And there have been no explicit records of significant donor rejection in several clinical trials for treatment [16]. Similarly characterized by chronic inflammation, tumors can be considered “wounds that never heal” [17]. MSCs are continuously recruited to tumors and become crucial components of the tumor microenvironment [18, 19].

Bioengineering methods, as a powerful approach to enhance the efficacy of MSCs, can expand the applications of MSC treatment, particularly in anti-cancer therapeutics. MSCs have been modified to deliver interferons, interleukins, anti-angiogenic agents, pro-apoptotic proteins, pro-drugs, or oncolytic viruses, to directly induce tumor apoptosis or activate immune cells to combat tumors [20–22]. Here, we summarize the mechanisms by which naïve MSCs are involved in tumor progression, as well as the applications and challenges of engineered MSCs in tumor treatment.

## MSCs as Trojan Horses

The tumor microenvironment generates an immunosuppressive niche, characterized by overrepresentation of immunosuppressive immune cells, continuous tissue remodeling, and a diverse array of cytokines, chemokines, and growth factors [23]. The anti-inflammatory immune state in the tumor microenvironment compromises healing processes [24]. A hallmark of all solid tumors is their heightened metabolic activity coupled with an inadequate oxygen supply [25]. During tumor progression, MSCs are mobilized and recruited to the tumor site and become tumor-associated MSCs in response to signals from growing tumors, thereby orchestrating the local immune microenvironment.

In 1955, Thomlinson and Gray proposed the concept of tumor hypoxia after examining histological sections of human epithelial tumors [26]. To this day, this concept has been repeatedly confirmed by scientists, who have reached a consensus that a hypoxic microenvironment is a common feature of most solid tumors [27, 28]. As transcription factors directly responding to the hypoxic environment, hypoxia-inducible factors (HIFs) are involved in fundamental cellular processes that promote tumor cell survival in the hostile hypoxic tumor microenvironment, such as glucose metabolism and angiogenesis. Thus, chaotic angiogenesis pathways and blood flow variations significantly hinder effective drug delivery to the tumor. Recently, scientists found that among the downstream target genes of HIF-1α, the concentration gradient of C-X-C motif ligand 12 (CXCL12) plays a pivotal role in recruiting MSCs expressing the CXCL12 receptor CXCR4 into the tumor microenvironment [29–31]. Regarding the direct effects of the hypoxic environment on MSCs, bone marrow, for example, is considered a tissue with limited oxygen supply, and bone marrow-derived MSCs (BM-MSCs) can be recruited to tumors that reach similarly low oxygen tension. Additionally, growth factors such as insulin-like growth factor 1 (IGF1), basic fibroblast growth factor (bFGF), vascular endothelial growth factor (VEGF), platelet-derived growth factor (PDGF), and transforming growth factor-β (TGF-β) can also recruit MSCs into the tumor environment [32–34]. Other tumor-derived factor like tumor necrosis factor-α (TNF-α) and interleukin-6 (IL-6) are also involved in MSC tumor-tropism by promoting endothelial adhesion and remodeling the cytoskeletal migration system [35, 36].

**Fig. 1:**
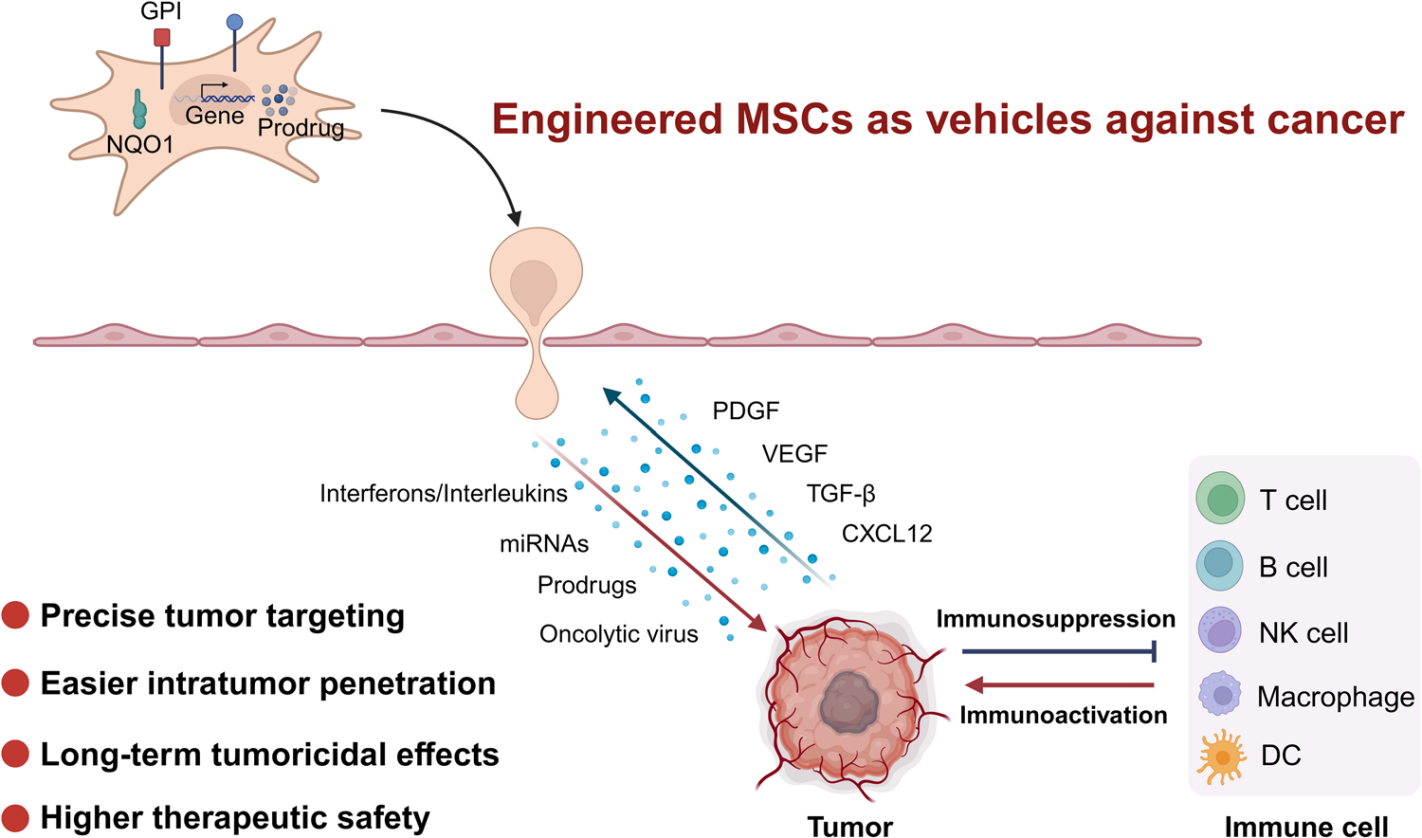
Engineered MSCs function as delivery vehicles in cancer therapy. MSCs are engineered to leverage their tumor-homing capabilities while exhibiting low immunogenicity, facilitating the targeted delivery of therapeutic proteins, microRNAs (miRNAs), prodrugs, and oncolytic viruses directly into the tumor microenvironment and activating antitumor immunity, which aims to enhance therapeutic efficacy while minimizing off-target effects. Key advantages of this approach include precise tumor targeting, improved intratumoral penetration, sustained tumoricidal effects, and higher therapeutic safety.

Their inherent ability to home to tumor sites can be harnessed for antitumor treatment, making them suitable for use as cellular therapies to deliver therapeutics directly to tumors. Genetically modified MSCs can function as ‘Trojan horses’ for cancer treatment. By homing to the tumor microenvironment, they can significantly enhance antitumor immunity or response to chemotherapy, while mitigating the toxicity associated with the systemic administration of high doses of cytokines or chemotherapeutic agents. Various strategies can be leveraged to further enhance the efficacy of MSC-based anticancer therapies and to tailor these therapies to the specific needs of different tumors and their microenvironments (Fig. 1).

## Engineered MSCs for Cancer Treatment

**Fig. 2:**
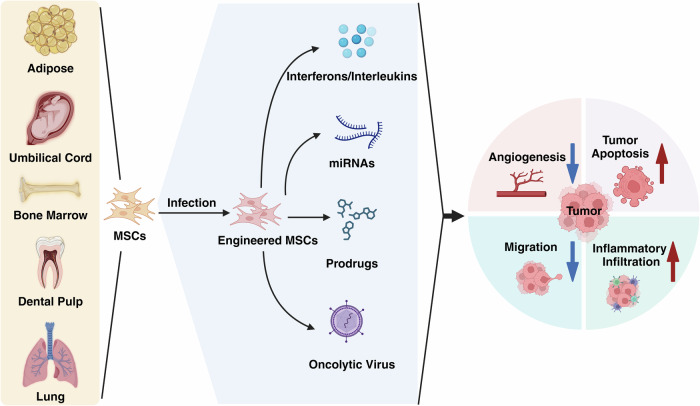
The multifaceted impacts of engineered MSCs on tumor progression. MSCs from different sources, including adipose tissue, umbilical cord, bone marrow, dental pulp, and lung, are engineered through viral infection and genetic modification to express therapeutic factors, such as interferons and interleukins, miRNAs, prodrugs, and oncolytic viruses. These engineered MSCs contribute to cancer therapy by modulating tumor biology—enhancing tumor apoptosis, inhibiting angiogenesis, reducing tumor cell migration, and modulating inflammatory infiltration.

Owing to the tumor tropism of MSCs, research efforts have focused on the genetic modification of MSCs via transfection to facilitate the overexpression of specific therapeutic agents [37, 38]. MSCs are readily amenable to genetic modification through viral vectors. This review will explore the engineering of MSCs through viral transfection-based modifications. The “ammunition” includes a spectrum of therapeutic proteins, microRNAs, prodrugs, chemotherapeutic drugs, suicide genes, and oncolytic viruses. MSCs, acting as efficient carriers, are equipped with these “munitions” which are subsequently delivered to the tumor site. These payloads, either by altering the immune cell landscape within the tumor microenvironment or exerting direct effects on tumor cells, play a crucial role in inhibiting tumor growth or directly inducing tumor cell death (Fig. 2).

### Delivery of therapeutic proteins

The tumor microenvironment is characterized by substantial infiltration of diverse immune cell populations, including T cells, B cells, natural killer (NK) cells, and macrophages. NK cells are innate immunocytes that directly kill tumors. However, in the highly metabolic environment of tumors, the accumulation of excessive lactate and depletion of amino acids (such as arginine and leucine) impair NK cell function [39]. In addition to metabolic constraints, the tumor microenvironment in hepatocellular carcinoma, breast cancer, and colorectal cancer contains PGE2 and indoleamine 2,3-dioxygenase (IDO), which inhibit the activation of DNAX accessory molecule-1 (DNAM-1) and NK cell activating receptors NKp44 and NKp30, thereby suppressing NK cell antitumor effects [40]. Metastatic tumors often evade T cell-mediated killing by downregulating MHC-I expression, thereby reducing antigen presentation and leading to immune therapy failure [41]. CD8^+^ T cells, recognized as the primary mediators of tumor eradication, frequently exhibit an exhausted phenotype characterized by the co-expression of PD-1 and TIM-3 [42, 43]. In addition to inherent factors contributing to T-cell exhaustion, immunosuppressive factors within the tumor microenvironment, such as IL-10 and TGF-β, can induce the transition of effector and memory T cells into an exhausted state [44, 45]. Researchers have suggested that reversing the exhausted phenotype in CD8^+^ T cells could potentially enhance tumor suppression. Among therapeutic proteins, cancer-prohibitory interferons and cytokines can achieve highly precise targeted delivery when transported by MSCs (Table 1).

**Table 1:** Examples of bioengineered MSCs with therapeutic proteins.

Engineered Agent	MSC source	Tumor Model	Mechanism	Refs
IFN-α	Mouse BM-MSCs	The mouse melanoma cell line B16F10	Induction of tumor cells and endothelial cell apoptosis	[49]
	Mouse BM-MSCs	The mouse melanoma cell line B16F0 and colon carcinoma tumor cell line MC38	Potentiate the cytotoxicity of CD8^+.^T cells and promote CD8^+^ T cell infiltration into tumor tissues	[50]
IFN-β	Human BM-MSCs	The mouse melanoma cell line A375SM	Inhibit the growth of malignant cells	[51]
	Mouse BM-MSCs	The mouse metastatic prostate cancer cell line TRAMP-C2	Increase the natural kill cell activity	[54]
	Mouse MSCs (The source was not mentioned)	The mouse breast cancer cell line 4T1	Inhibit STAT3 signaling; reduce pulmonary and hepatic metastases	[55]
	Mouse MSCs (The source was not mentioned)	The mouse glioblastoma multiforme cell line CT-2A and GL261	Enhance selective post-surgical infiltration of CD8^+^ T cells; induce cell-cycle arrest in tumor cells	[137]
IFN-γ	Human BM-MSCs	The human neuroblastoma cell lines CHLA-255 and CHLA-20	Promote proinflammatory polarization of macrophages	[38]
IL-2	Rat BM-MSCs	The rat glioma cell line 9 L	Inhibit tumor growth	[61]
	Mouse BM- MSCs	The mouse melanoma cell line B16F0	Develop CD8^+^ T cell-mediated tumor-specific immunity and delay tumor growth	[62]
	Mouse BM-MSCs	The mouse melanoma cell line B16F0, colon carcinoma MC38 and CT26 and breast cancer cell line 4T1	Rejuvenate exhausted TILs by expanding PD1^+^TIM3^−^CD8^+^ T cells and reinvigorating cytotoxic activities	[125]
IL-12	Human UB-MSCs	The human glioma cell line GL261	Enhance IFN-γ secretion and T cell infiltration as well as anti-angiogenesis	[64]
	Mouse BM-MSCs	Primary and metastatic B16F10 melanomas	Decrease vascular density and increase the number of anticancer proinflammatory macrophages and CD8^+^ cytotoxic T lymphocytes in tumors	[65]
IL-18	Human UC-MSCs	The human breast cancer cell line MCF-7 and HCC1937	Inhibit tumor cell growth, migration and invasion in vitro	[67]
IL-21	Human UB-MSCs	The human primary ovarian cancer cell	Increase IFNγ-secreting splenocyte numbers and natural killer cytotoxicities	[68]
	Human UC-MSCs	The human ovarian cancer cell line SKOV-3	Increase the expression of NKG2D and MIC-A molecules in the tumor tissues, and inhibit SKOV3 ovarian cancer growth by downregulate β-catenin and cyclin-D1 in the tumor tissues	[69]
Trail	Human UB- MSCs	The human glioma cell line U-87MG, U-251MG and A172	Not elucidated	[70]
	Human AD-MSCs	The human lung cancer cell line H460	Increase tumor apoptosis	[71]
IL-12+Trail	Mouse BM-MSCs	The mouse lymphoma cell line L5178Y	Increase tumor apoptosis	[138]

*MSCs* mesenchymal stem/stromal cells, *BM* bone marrow, *UB* umbilical cord blood, *UC* umbilical cord, *AD* adipose tissue, *IFN* interferon, *IL* interleukin, *STAT3* signal transducer and activator of transcription 3, *TIL* tumor-infiltrating lymphocyte.

#### Interferons

Type I and type II interferons are expressed under physiological conditions and are important regulators of immunity and inflammation [46]. On the one hand, type I interferons are known for their pivotal role in antiviral immune responses [47]. Type I interferons promote antigen presentation and NK cell function, while inhibiting proinflammatory pathways and cytokine production. They activate the adaptive immune system, promoting high-affinity antigen-specific T cell and B cell responses and immunological memory [48]. An increasing body of evidence suggests that exogenous type I interferons exhibit antitumor efficacy when administered at specific tumor sites. Ren et al. first genetically engineered MSCs to overexpress IFN-α (IFNα-MSCs) and observed their ability to suppress tumor growth through the induction of apoptosis in both tumor cells and tumor-associated endothelial cells [49]. Furthermore, IFNα-MSCs stimulated the production of CXCL10 in tumor cells, upregulated the expression of granzyme B (GZMB) in CD8^+^ T cells, and increased the number and cytotoxic activity of infiltrating CD8^+^ T cells [49, 50]. Similarly, IFNβ-MSCs can directly kill tumor cells independent of the immune system [51]. More importantly, IFNβ-loaded MSCs can initiate a robust antitumor immune response in the tumor microenvironment. IFN-β plays a regulatory role in the expression of TNF-α and IL-12 in peripheral blood monocytes [52]. Additionally, IFN-β modulates the expression of chemokines, including CXCL10, in macrophages [53]. Furthermore, IFNβ-MSCs can enhance NK cell activities [54]. Another study demonstrated that this cell-mediated immunity increases the numbers of splenic mature dendritic cells (DCs) and decreases the numbers of Treg cells [55].

IFN-γ, a type II interferon, was initially believed to possess antitumor effects; however, subsequent research revealed that IFN-γ promotes the expression of inhibitory molecules such as programmed death-ligand 1 (PD-L1), PD-L2, IDO1, inducible nitric oxide synthase (iNOS), FAS, and FAS ligand (FASL), all of which limit antitumor immunity [56]. In earlier studies, we found that IFN-γ significantly enhances the immunosuppressive capacity of MSCs in immune regulation [57, 58]. Although IFN-γ, as a stimulatory factor for proinflammatory macrophages, can yield promising antitumor effects, caution should be exercised in utilizing IFNγ-MSCs for tumor immunotherapy [38]. Additionally, IFNs can enhance the expression of surface MHC molecules, including in MSCs, and then increase the processing and presentation of tumor-specific antigens, thus facilitating T-cell recognition and cytotoxicity [59, 60]. Given that IFNs are classic cytokines known to induce MSC immunosuppression, caution is warranted on IFNs-overexpression MSCs in cancer therapy.

#### Interleukins

Engineered MSCs overexpressing cytokines demonstrate potent antitumor effects in various cancer models through mechanisms such as reinvigoration of exhausted tumor-infiltrating lymphocytes, augmentation of cytotoxic T cell and NK cell activities, reduction of tumor vascularization, and induction of tumor cell apoptosis and necrosis. MSCs overexpressing IL-2 (IL2-MSCs) exhibited inhibitory effects on glioma and melanoma in murine models, potentially mediated through the involvement of CD8^+^ T cells within the tumor microenvironment [61, 62]. These MSCs were engineered with the dual objectives of enhancing the homing capacity of MSCs to tumor sites and optimizing the interaction between IL-2 and its respective receptors. In this context, IL2-MSCs reinvigorated exhausted tumor-infiltrating lymphocytes (TILs) by amplifying the population of PD1^+^TIM3^-^CD8^+^ T cells, while concurrently diminishing the frequency of terminally exhausted T cells and reigniting their cytotoxic functionality [62]. IL-12, a potent proinflammatory cytokine, has garnered extensive attention as a prospective immunotherapeutic agent for cancer. However, initial clinical trials showed that systemic delivery of IL-12 led to dose-limiting toxicities [63]. Thus, local administration of IL12-MSCs demonstrated excellent therapeutic effects in glioma and melanoma. These mechanisms may involve the augmentation of IFN-γ secretion, reduction of vascular density, and increase in proinflammatory macrophages and CD8^+^ cytotoxic T lymphocytes (CTLs) [64, 65]. IL-18 can stimulate T cells and NK cells, prompting them to secrete IFN-γ and granulocyte-macrophage colony-stimulating factor (GM-CSF). IL-18 enhances the cytolytic activity of NK cells, promotes the proliferation of T cells, activates CD8^+^ CTLs, and acts as a chemoattractant for immature DCs. This recruitment is a pivotal event capable of eliciting robust immune responses [66]. Indeed, IL18-overexpressing human UC-MSCs (IL18-hUCMSCs) effectively curtailed tumor cell proliferation, migration, and invasion in the in vitro experiments [67]. Similarly, the introduction of IL-21 into MSCs (IL21-MSCs) substantially augmented NK cytotoxic activity by elevating the secretion of secondary cytokines, including IFN-γ and TNF-α. This effect led to the induction of tumor cell necrosis, apoptosis, and vascular hemorrhage [68, 69]. Additionally, TRAIL-engineered MSCs also showed antitumor effects [70, 71]. These approaches highlight the potential of cytokine-engineered MSCs as innovative and effective strategies for cancer immunotherapy, emphasizing the importance of localized cytokine delivery to minimize systemic toxicities and enhance therapeutic outcomes.

### Delivery of miRNA

The interaction between MSCs and tumors can be mediated through extracellular vesicles (EVs). A diverse subset of EVs, especially exosomes, has gained significant attention in recent times [72]. Notably, exosomes, which are ubiquitously present in various body fluids, are characterized by the inheritance of their parental molecular and genetic profiles [73]. Remarkably, exosomes can traverse the blood-brain barrier and reach virtually every region of the human body, an attribute that circumvents a limitation associated with conventional cell-based therapies [74]. MicroRNAs (miRNAs) are non-coding RNA molecules known for their diverse roles in various biological processes, primarily through their post-transcriptional regulation of gene expression [75]. The occurrence and progression of tumors are often associated with the up-regulation or down-regulation of various miRNAs. The extensive study of the myriad miRNAs linked to tumorigenesis remains an active area of research. The exosome-derived secretome from MSCs holds significant promise for advancing tumor therapy (Table 2).

**Table 2:** Examples of bioengineered MSCs with miRNA.

Engineered agent	MSC source	Tumor Model	Mechanism	Refs
miR-101/ miRNA-101-3p	Human BM-MSCs	The human oral cancer cell lines	Inhibit COL10A1 and inhibit proliferation, invasion, and migration of oral cancer cells	[76]
	Human AD-MSCs	The human osteosarcoma cell lines	Suppress osteosarcoma invasiveness and metastasis partially through regulation of BCL6	[77]
miRNA-125a	Non-mentioned	The mouse nasopharyngeal carcinoma cell lines	Suppress neonatal blood vessel formation	[84]
miRNA-145	Human AD-MSCs	The human breast cancer cell lines	Inhibit the invasion ability of metastatic breast cancer cell lines and prevent TGFβ-dependent tumor formation	[139]
miR-374c-5p	Human BM-MSCs	The human hepatocellular carcinoma cell lines	Inhibit tumor metastasis via inhibiting LIMK1 and Wnt/β-catenin signaling pathway	[88]
miRNA-424	Mouse BM-MSCs	The human ovarian cancer cell lines and human umbilical vein endothelial cells	Repressed the proliferation, migration, and invasion of ovarian cancer cells, with a reduction in the expression of VEGF and VEGFR; suppress tumorigenesis and angiogenesis of ovarian tumors in vivo	[89]
miR-655-3p	Human UC-MSCs	The human carcinoma cell lines and human esophageal epithelial cell lines	Inactivation of HIF-1α via a LMO4/HDAC2-dependent mechanism	[90]

*MSCs* mesenchymal stem/stromal cells, *BM* bone marrow, *AD* adipose tissue, *UC* umbilical cord, *COL10A1* collagen type X alpha 1 chain, *BCL6* B cell leukemia/lymphoma 6, *TGFβ* transforming growth factor beta, *VEGF* vascular endothelial growth factor, *VEGFR* vascular endothelial growth factor receptor, *HIF-1α* hypoxia inducible factor 1 subunit alph, *LMO4* LIM domain only 4, *HDAC2* histone deacetylase 2.

The expression of miR-101 is significantly downregulated across a broad spectrum of malignancies. The introduction of miR-101 into MSCs (miR101-MSCs) demonstrated enhanced therapeutic efficacy in osteosarcoma and oral cancer [76, 77]. MiR-125a can directly target specific oncogenes, as reported in various studies [78]. Moreover, miR-125a has been consistently observed to be downregulated in ovarian cancer [79], cervical cancer [80], breast cancer [81], gastric cancer [82], and medulloblastoma [83]. Recently, the treatment of nasopharyngeal carcinoma cells with exosomes overexpressing miR-125a from miR125a-MSCs has been shown to be efficacious in suppressing vasculogenic mimicry formation in both in vitro and in vivo models [84]. The miR-34 family, directly regulated by the tumor suppressor p53, is recognized as a crucial component in tumor suppression. However, methylation of its promoter region results in miR-34 downregulation, contributing to the pathogenesis of ovarian and colorectal cancers [85, 86]. Epithelial-mesenchymal transition (EMT) is often associated with the downregulation of miR-374c-5p during cancer development [87]. The overexpression of miR-374c-5p in MSCs inhibited EMT in hepatocellular carcinoma through the LIMK1-Wnt/β-catenin axis, halting the occurrence and development of liver cancer [88]. The transcriptional regulator MYB showed heightened expression in ovarian cancer, potentially regulated by reduced levels of miR-424. Exosomes secreted by MSCs overexpressing miR-424 significantly impacted the onset and progression of ovarian cancer, holding promise for clinical applications [89]. Similarly, MSCs with elevated miR-6553p expression have been demonstrated to be effective in esophageal squamous cell carcinoma (ESCC) [90]. Additionally, numerous miRNAs undergo dysregulation in tumorous conditions, underscoring the potential of EVs/exosomes as innate vesicular entities acting as highly effective and promising drug delivery systems for precise and targeted tumor therapy. Overall, engineering MSCs to overexpress specific miRNAs offers an effective approach for cancer treatment, and has therapeutic potential in various cancers by inhibiting tumor progression, promoting apoptosis, and preventing vasculogenic mimicry and EMT through miRNA-mediated modulation of oncogenic pathways and gene expression.

### Drug delivery

#### Prodrugs

Cancer treatment using prodrug delivery methodologies began in the early 1980s [91]. Previous therapeutic approaches were involved in introducing a non-toxic enzyme gene under a tumor-specific promoter. After the administration of a non-toxic prodrug, these enzymes catalyze the conversion of the prodrug into toxic metabolites, leading to programmed cell death in tumor [92, 93]. Prominent systems in this domain include the herpes simplex virus thymidine kinase/ganciclovir (HSV-TK/GCV) system [94] and the cytosine deaminase/5-fluorocytosine (CD/5-FC) system [95]. However, in solid tumors, gaining access to the innermost tumor cells is a formidable challenge [96]. Tumor proliferation is often rapid and nearly uncontrolled. While the prodrug delivery system can trigger apoptosis, it may also generate a ‘bystander effect’ that affects adjacent cells, potentially diminishing its efficacy against cancer cells [97]. Therefore, this type of clinical treatment has seen slow progress. Exosomes from MSCs have been studied as delivery vehicles [98]. The mRNA of suicide genes carried by exosomes from engineered MSCs was internalized by recipient tumor cells, triggering tumor cell death in the presence of a prodrug [98] (Table 3).

**Table 3:** Examples of bioengineered MSCs with drugs.

Engineered agent	MSC source	Tumor Model	Mechanism	Refs
Gemcitabine	Human DP-MSCs	The human pancreatic carcinoma	Suppress tumor growth	[113]
Paclitaxel	Human GP-MSCs	The human pancreatic adenocarcinoma, glioblastoma multiforme, human mesothelioma and human squamous cell carcinoma	Suppress tumor growth	[114]
Doxorubicin	Human BM-MSCs	The human glioma	Suppress tumor growth	[140]
	Human BM-MSCs derived exosomes	The human osteosarcoma	Suppress tumor growth	[141]
Cytosine deaminase/5-fluorocytosine	Human BM-MSCs	The human osteosarcoma	Suppress tumor growth	[108]
	Mouse AD-MSCs	The mouse lung carcinoma	Suppress tumor growth	[109]
	Human BM-MSCs	The human glioma	Suppress tumor growth	[110]
Herpes simplex virus thymidine kinase/ganciclovir	Human BM, DP, UC, blood platelets and other tissue derived MSCs	The human glioma	Suppress tumor growth	[101]
	Human AD-MSCs	The human primary glioblastoma	Suppress tumor growth	[103]
	Mouse BM-MSCs	The human prostate adenocarcinoma	Suppress tumor growth	[102]

*MSCs* mesenchymal stem/stromal cells, *DP* dental pulp, *GP* gingival papilla, *BM* bone marrow, *AD* adipose tissue, *UC* umbilical cord.

The neurotropism of HSV makes it a widely used agent in the treatment of glioma [99]. This enzyme has a high affinity for ganciclovir and generates triphosphorylated ganciclovir (GCV) when catalyzed by endogenous cellular enzymes [100]. During DNA synthesis, GCV undergoes three phosphorylation steps and is subsequently integrated into DNA strands, impeding DNA chain elongation and ultimately resulting in cell death. Exosomes loaded with herpes simplex virus thymidine kinase (HSV-TK) from MSCs (HSV-TK/GCV) are internalized by tumor cells. In the presence of the prodrug GCV, these exosomes facilitate the intracellular conversion of ganciclovir into GCV triphosphate, resulting in the death of the recipient tumor cells [101, 102]. This system demonstrates a potent inhibitory effect on glioma and is regarded as a cell-specific drug delivery method that acts within tumor cells [101–103].

Cytosine deaminase (CD) is an enzyme found in bacteria and fungi but absent in mammalian cells [92]. 5-Fluorouracil (5-FU) can passively diffuse through cellular membranes, thereby inflicting cytotoxicity in cells adjacent to those expressing CD [92, 104]. 5-FU is enzymatically converted to 5-fluoro-2’-deoxyuridine-5’-monophosphate (5-FdUMP) or phosphorylated to 5-fluorouridine-5’-triphosphate (5-FUTP) [105, 106]. These metabolites derived from 5-FC can be incorporated into both RNA and DNA, leading to the inhibition of nuclear processing and inflicting direct damage on newly synthesized DNA strands [106]. While prodrug delivery systems utilizing CD and 5-FC have shown promise in treating certain tumor types, the direct delivery of therapeutic agents to cancer cells within solid tumors is often hindered by irregular vascularization and tight tissue architecture [107]. Engineered MSCs present a plausible solution for converting the prodrug 5-FC into its toxic counterpart 5-FU. Indeed, studies have demonstrated that MSCs engineered with CD/5-FC successfully inhibit the growth of osteosarcoma [108], lung carcinoma [109], and glioma [110]. Overall, prodrug delivery systems in cancer treatment leverage engineered MSCs and exosomes to convert non-toxic prodrugs into toxic metabolites within tumor cells, presenting a targeted approach to induce tumor cell apoptosis despite challenges in treating solid tumors.

#### Chemotherapy drugs

Chemotherapy is a conventional and widely employed treatment modality. Chemotherapy often carries significant risks and can lead to severe consequences for cancer patients, resembling a balance of “either you kill me or we both die.” Its non-selective nature often results in collateral damage to normal tissues. Therefore, achieving targeted and localized drug delivery has become a primary objective in the field of chemotherapy [111]. Chemotherapeutic agents such as gemcitabine (GCB), paclitaxel (PTX), and doxorubicin (DOX) remain widely used in cancer treatment. In the quest for more effective and less toxic cancer treatments, the engineering of MSCs for targeted drug delivery has emerged as a promising strategy.

GCB is classified as a pyrimidine nucleotide analogue and falls within the category of cancer antimetabolites. Metabolites of GCB inhibit DNA synthesis and prevent cell cycle transition from the G1 phase to the S phase, ultimately leading to apoptosis [112]. Recent evidence showed that GCB loaded within extracellular vesicles derived from dental pulp-derived MSCs (DPMSC-sEV) was as effective as the pure drug and efficiently inhibited pancreatic carcinoma growth in vitro [113]. PTX is a widely used chemotherapy drug with a well-established reputation in clinical practice. It functions by inhibiting cell mitosis through promoting tubulin polymerization and inhibiting tubulin depolymerization. However, delivering PTX specifically to tumor sites has been a significant challenge, prompting researchers to exploit nanometer-scale delivery methods. Despite these advancements, the limited tumor-targeting capacity of these methods has hindered further development in clinical trials. Exposed to high concentrations of PTX in vitro, MSCs can load and deliver the drug via EVs. These results showed that exosomes generated by gingival MSCs can be an ideal vector, delivering high drug concentrations in a minimal volume [114]. This method has been proven effective in pancreatic carcinoma, glioblastoma, mesothelioma, and squamous cell carcinoma [114]. Traditional chemotherapy agents demonstrate enhanced efficacy with reduced side effects when delivered through MSC-derived extracellular vesicles, marking a significant advance in achieving localized treatment. This approach not only addresses the inherent non-selectivity of conventional chemotherapy, which often harms healthy tissues, but also leverages the natural tumor-homing capabilities of MSCs, offering a novel pathway for precision oncology.

### Delivery of oncolytic virus

Oncolytic virotherapy, using both genetically modified and naturally occurring viruses, offers a targeted approach to cancer treatment by selectively infecting and lysing tumor cells. Using MSCs as vectors to deliver oncolytic viruses directly to tumors enhances this specificity, overcoming challenges of non-specific retention and systemic toxicity. This strategy has shown promise in treating malignancies such as glioma, hepatocellular carcinoma, and metastatic melanoma, utilizing MSCs’ tumor-homing capabilities and the ability of viruses to induce potent immune responses against cancer cells (Table 4).

**Table 4:** Examples of bioengineered MSCs with oncolytic virus.

Engineered agent	MSC source	Tumor Model	Mechanism	Refs
Oncolytic adenovirus	Human MSCs	Human glioblastoma and human lung carcinoma	Suppress tumor growth	[120]
	Human BM-MSCs	The human non-small lung carcinoma and human hepatocarcinoma	Suppress tumor growth	[121]
Oncolytic measles virus	Human AD-MSCs	The human ovarian cancer	Suppress tumor growth	[120]
	Human BM-MSCs	The human hepatocellular carcinoma	Suppress tumor growth	[123]
Oncolytic herpes simplex virus	Human and mouse MSCs	The human melanoma	Suppress tumor growth	[122]
Oncolytic myxoma virus	Human AD-MSCs	The human glioblastoma	Suppress tumor growth	[142]
Oncolytic newcastle disease virus	Human BM, AD, UC-MSCs	The human glioma	Suppress tumor growth	[143]

*MSCs* mesenchymal stem/stromal cells, *BM* bone marrow, *AD* adipose tissue, *UC* umbilical cord.

The most widely utilized oncolytic virus is the oncolytic adenovirus. Due to the natural affinity of adenoviruses for liver and other organ cells [115], modifications predominantly focus on capsid protein engineering to manipulate viral tropism, to redirect viral infection, to reduce non-specific viral retention in non-tumor locations, and to optimize cellular delivery [116–118]. MSCs are ideal vector cells with intrinsic tumor tropism [119], carrying viruses across host defenses and directing them to tumors. Oncolytic viruses have exhibited remarkable therapeutic efficacy in treating malignant intracranial glioma and hepatocellular carcinoma [120, 121]. This therapeutic effect is characterized by heightened immunogenicity and reduced blood circulation duration, addressing non-specific hepatic sequestration and associated liver toxicity. A recent study showed that HSV-armed MSCs were effective in tracking and killing metastatic melanoma cells in the brain [122]. The oncolytic measles virus presents a strong cytopathic effect. Oncolytic measles virus, delivered via MSCs, may inhibit tumor growth through xenogeneic cell fusion. Existing studies have shown that MSCs carrying oncolytic measles virus have a strong inhibitory effect on liver cancer [123] and ovarian cancer [124]. However, further research is needed to fully understand the mechanisms of selectivity and action of oncolytic viruses in this context.

## Optimization of Engineered MSCs

The use of engineered MSCs and their exosomes for targeted tumor therapy represents a significant advancement in cancer treatment, providing a platform for the controlled release of therapeutic agents. MSCs have a wide range of sources and are relatively easy to obtain, with lower ethical risks compared to other cell types. For example, MSCs engineered to deliver IL-2 mutein dimer to tumor-infiltrating T cells exhibit capacity to reinvigorate exhausted CD8^+^ T cells, making it a more potent antitumor therapeutic agent [125]. Concurrently, MSCs were engineered with a hypoxia-inducible promoter and NAD(P)H: quinone oxidoreductase 1 (NQO1), responsible for regulating the production of IL-2 within the tumor microenvironment. This modification not only enhances MSC survival in the challenging tumor microenvironment but also renders these cells more susceptible to β-lap-mediated cytotoxicity, offering a new therapeutic paradigm. Additionally, due to the complexity of the tumor microenvironment and the heterogeneous expression patterns of solid tumor antigens, any strategy relying solely on a single effector targeting one antigen may not achieve complete tumor eradication [126]. The team of M. Suzuki presents a novel binary vector containing OAd and HDAd (a helper-dependent Ad), which directly kills tumors, activates the immune response through IL-12, and facilitates PD-L1 immune checkpoint therapy [127, 128]. This implies that the vector potential of MSCs can be augmented through the integration of oncolytic virus therapy, cytokine therapy, and immune checkpoint therapy.

Although engineered MSC exosomes exhibit the drawbacks of limited circulatory lifespan and suboptimal targeting efficiency [129], their transformation is undergoing significant progress through surface alterations, peptide insertion, and chemical modifications. The incorporation of glycosylphosphatidylinositol (GPI) serves to protect exosomal surface proteins against proteolytic degradation and facilitates the targeted delivery of exosomes to tumor tissues [130]. The specificity and circulation time of exosomes modified with PEGylated liposomes were significantly prolonged [131]. Diverse tumor cells exhibit distinct antigen profiles, some of which are exclusive to tumor cells and others characteristic of specific tumor types. Therefore, incorporating antibodies that can selectively bind to tumor antigens on exosome surfaces is a promising strategy.

The immunoregulatory function of MSCs is highly plastic. After being recruited by chemokines in the tumor microenvironment, the immunosuppressive niche initially created by MSCs is reversed by the factors they produce following transformation [132]. Inflammatory cytokines upregulate MSCs to express PD-L1 or induce the expression of PD-L1 in tumors, thereby conferring immunosuppressive properties [133]. This mechanism offers potential for combination with immune checkpoint blockade therapies [134, 135]. In contrast, certain tissue-derived MSCs may exert immunosupportive effects on immune cells [136]. Thus, a better understanding of the plasticity of immunoregulation by MSCs will help optimize strategies for MSC applications in tumor treatment. Overloading IFNs or other drugs inevitably lead to induced intratumoral IFN production. Consequently, the precise role of upregulated MSC histocompatibility complex in the tumor microenvironment, whether it operates in a “hit-and-run” manner or through “long-lasting release,” remains unclear. Additionally, investigating the alterations in the tumor microenvironment following engineered MSC treatment will provide further evidence to support the combination of immune checkpoint blockade therapy and the clinical application of MSCs [50, 125, 128].

**Fig. 3:**
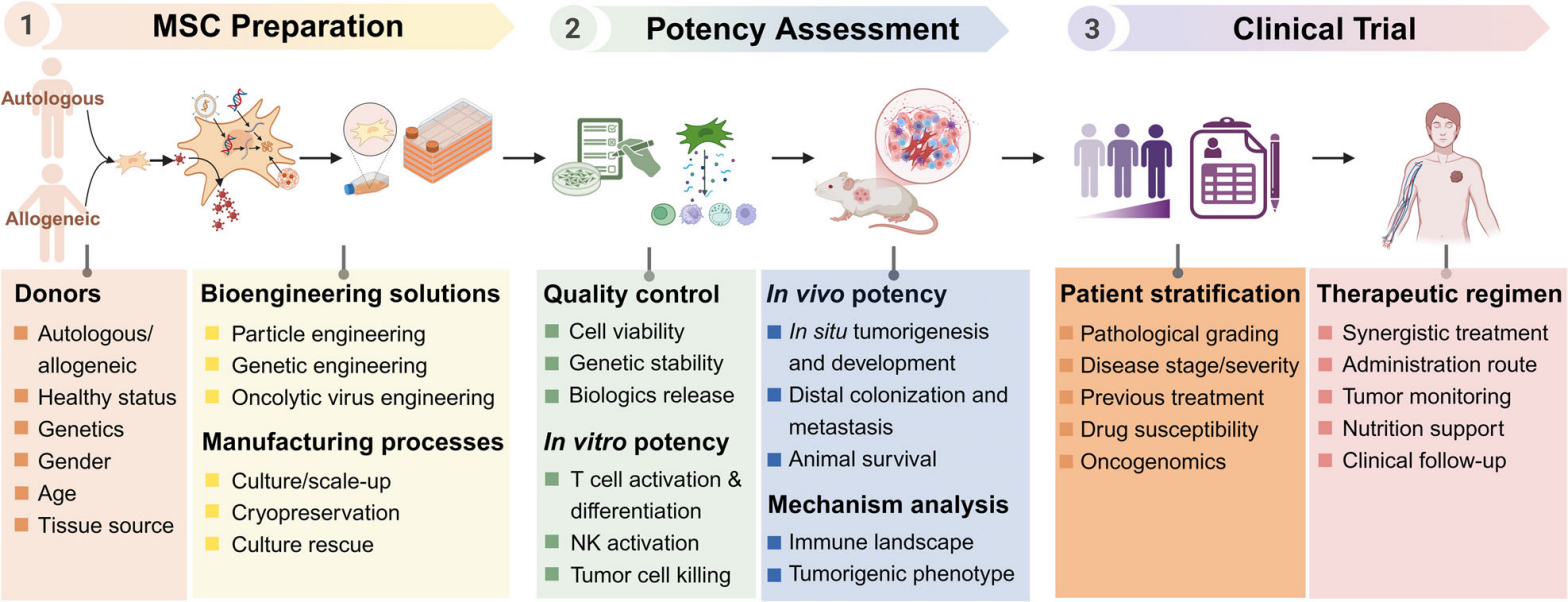
The translational pathway of engineered MSCs from laboratory research to clinical application. Initially, the preparation and modification of MSCs involve meticulous donor selection, considering factors such as health status, genetic background, sex, age, and tissue origin. Subsequently, bioengineering approaches—such as particle engineering, genetic modifications, and oncolytic virus incorporation—are integrated into standardized manufacturing processes to produce engineered MSCs. In the intermediate stage, rigorous quality control measures and potency assessments are conducted. These steps include monitoring cell viability, ensuring genetic stability, and evaluating the release of biologic factors, along with conducting in vitro and in vivo efficacy tests. Characterization of the immune microenvironment and tumorigenic phenotypes further elucidates the therapeutic potential and underlying mechanisms of action. Finally, during clinical trials and application, patient stratification is performed based on factors such as pathological grading, disease stage and severity, prior treatments, drug susceptibility, and oncogenomic profiles. This stratification enables the formulation of personalized therapeutic regimens, incorporating synergistic treatment strategies, tailored administration routes, continuous tumor monitoring, nutritional support, and clinical follow-up. Together, these steps enable the safe, effective, and precise translation of engineered MSC therapies from bench to bedside.

Current therapeutic strategies involve isolating MSCs in vitro, followed by the establishment of stable cell lines to enable large-scale expansion. After validation in animal models, these approaches hold the potential to advance into clinical trials (Fig. 3). Despite these advances, MSCs and MSC-derived exosomes have demonstrated limitations in targeting efficiency and therapeutic potential. Further investigations are needed to fully harness the potential of MSC-based therapies in oncology for clinical applications.

## Conclusions

The exploration and utilization of MSCs have marked a significant advancement in cancer therapy, bridging decades of research from their initial discovery in bone marrow stroma to their identification across various tissues. MSCs have demonstrated remarkable versatility, not only in their differentiation potential across mesenchymal and non-mesodermal cell types but also in their ability to interact with and modulate the tumor microenvironment. The development and application of engineered MSCs have opened new avenues for targeted cancer treatment, utilizing their low immunogenicity and innate tumor tropism. Another important aspect is that engineered MSCs secrete cytokines that increase the number of memory T cells in tumor-draining lymph nodes, thereby enhancing long-term immune surveillance [50]. Moreover, these cells elevate the expression of tumor PD-L1 or T cell PD-1 when combined with immune checkpoint inhibitors, a strategy that has shown superior outcomes in tumor therapy [50, 125]. Engineered MSCs, through viral transfection or other modification techniques, have been tailored to deliver a wide array of therapeutic agents—including therapeutic proteins, miRNAs, prodrugs, chemotherapy drugs, and oncolytic viruses—directly to the tumor site. Innovations in exosome modification, surface alterations, and the incorporation of specific targeting moieties aim to refine the delivery of therapeutic agents and increase the precision of MSC-based therapies. Despite the promising outcomes of MSC-based therapies in preclinical research and clinical trials, challenges remain. For instance, the heterogeneity of MSCs derived from different tissue sources can lead to unpredictable therapeutic outcomes [136]. Additionally, it is essential to assess whether the homing ability and proliferative potential of genetically modified MSCs remain consistent after in vitro expansion, compared to their pre-modification state. Furthermore, it is crucial to examine whether external signaling cues or intercellular communication could trigger inappropriate differentiation of these cells. Therefore, it is vital to establish standardized protocols and rigorous evaluation criteria for the application of engineered MSCs in cancer therapy, aiming to effectively eradicate tumors while minimizing the risk of adverse effects.

The journey of MSCs from discovery to therapeutic agents in cancer treatment exemplifies the dynamic interplay between scientific innovation and clinical application. While significant progress has been made, ongoing research and development are crucial to fully harness the potential of engineered MSCs in revolutionizing cancer therapy, establishing it as a promising frontier in precision oncology.
